# GSK3β activation is a key driver of resistance to Raf inhibition in BRAF mutant melanoma cells

**DOI:** 10.18632/oncotarget.28711

**Published:** 2025-04-04

**Authors:** Diana Crisan, Susanne Schatz, Heidi Hainzl, Fang Zhao, Annette Paschen, Maria Crisan, Adrian Baican, Karin Scharffetter-Kochanek, Abhijit Basu

**Affiliations:** ^1^Clinic of Dermatology and Allergic Diseases, University Hospital Ulm, Ulm, Germany; ^2^Experimental Laboratories of the Department of Dermatology and Allergic Diseases, Ulm, Germany; ^3^Department of Dermatology, University Hospital Essen, University Duisburg-Essen, Essen and German Cancer Consortium (DKTK), partner site Essen/Düsseldorf, Essen, Germany; ^4^Department of Dermatology, “Iuliu Hatieganu” University of Medicine and Pharmacy Cluj-Napoca, Romania

**Keywords:** BRAF melanoma resistance mechanisms, GSK3 β, BRAF mutation

## INTRODUCTION

Adaptive metabolic reprogramming with oxidative phosphorylation, anaerobic glycolysis and autophagy are major mechanisms of both *de novo* and acquired resistance to inhibitors of the Mitogen Activated Protein Kinase pathway (MAPKi) in melanoma [1]. MAPKi resistant melanoma cells activate alternative pathways like the PI3K/mTOR growth pathway or induce expression and activation of transcription factors like CREB which further promote tumor progression [2–4]. So far, the molecular mechanism of how CREB-activated genes may drive MAPKi-resistance of melanoma have not been investigated.

We here found that overexpression of CREB in A375 BRAF-mutant melanoma cells resulted in upregulation of genes involved in glucose metabolism including Glycogen synthase kinase 3 beta (GSK3β), suggesting a novel role for CREB-activated pathways in metabolic rewiring, which may be required for the development of MAPKi resistance in melanoma (Supplementary Figure 1).

GSK3β is a ubiquitously expressed serine/threonine kinase with essential role in glycogen metabolism, transcriptional regulation and cell survival. Though its oncogenic role is poorly understood, first indirect evidence showed that GSK3β activation protected melanoma cells from apoptosis, whereas its inhibition prevented mouse melanoma cell growth both *in vivo* and *in vitro* [5].

**Figure 1:**
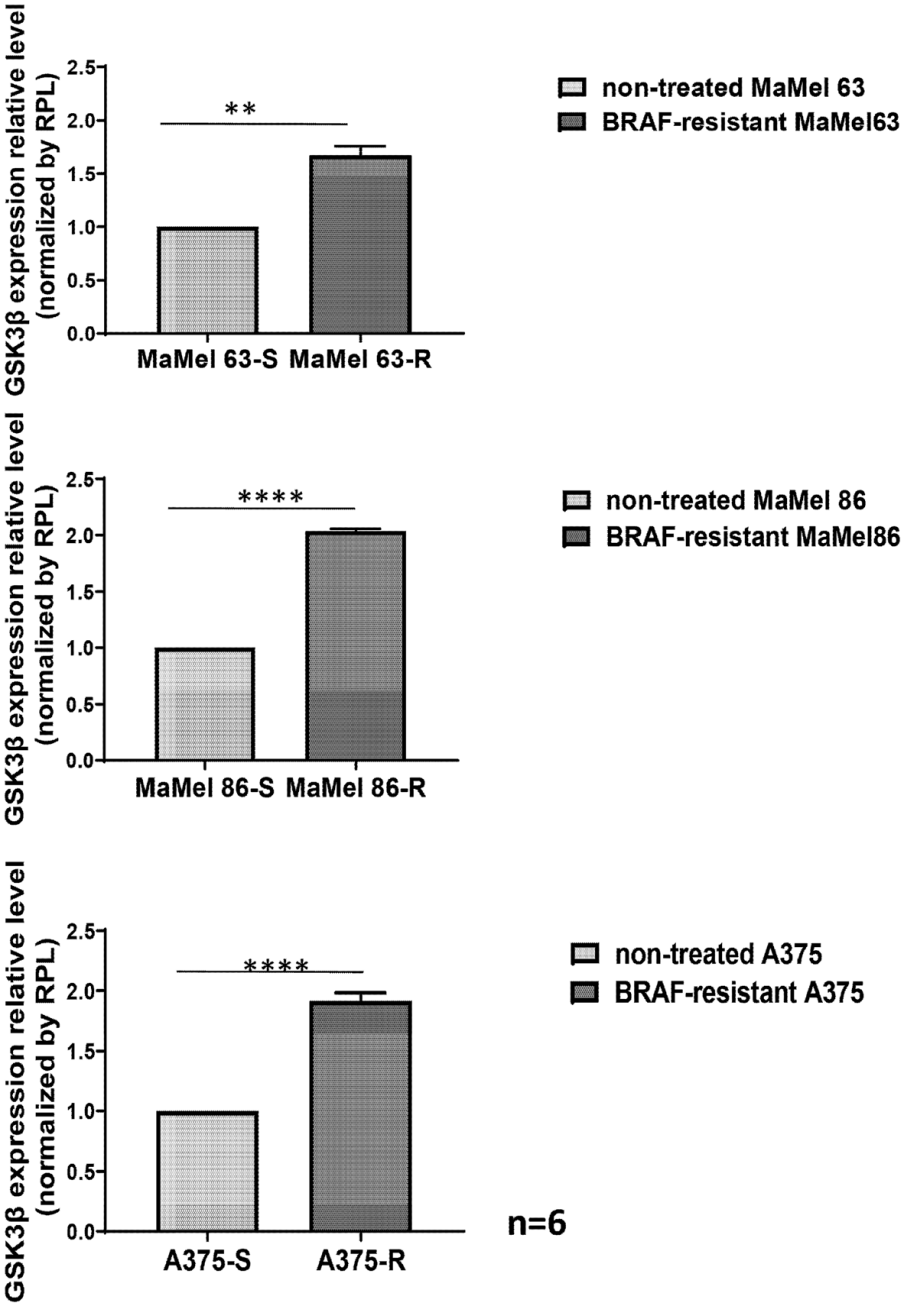
Acquired BRAF resistance leads to increased GSK3β expression. GSK3β expression was assessed by qPCR (primer sequence: 5’-gac taa ggt ctt ccg acc cc-3’ for; 5’-aag agt gca ggt gtg tct cg-3’ rev) from three melanoma cell lines before and after the development of BRAF resistance. A375 BRAF-mutated melanoma cells developed a resistant state after 8 weeks of continuous treatment with the BRAFi Dabrafenib at a concentration of 1 μM twice a week. Two independent melanoma models of paired BRAFi-sensitive and BRAFi-resistant cells Ma-Mel-63a and Ma-Mel-86c were used to confirm the findings. Bars indicate GSK3β expression levels normalized by RPL13 and expressed as mean log2 fold change ± SD. ^**^ *p* < 0.001, ^****^ *p* < 0.00001 using one-way ANOVA.

Here we set out to investigate the impact of GSK3β on the development of BRAF inhibitor (BRAFi) resistance in melanoma. For this purpose, melanoma cell lines bearing a defined BRAF mutation (A375 BRAF-V600E) were treated with the small molecule BRAF inhibitor Dabrafenib (GSK2118436) until developing resistance. GSK3β expression and activation in A375 BRAF-V600E mutant melanoma cells were assessed prior to BRAFi treatment, in BRAFi-sensitive cells and after developing BRAFi resistance at RNA and protein levels. We observed a significant increase in GSK3β mRNA expression in A375 BRAF-mutated melanoma cells during the development of BRAF resistance (Supplementary Figure 2). BRAFi-resistant melanoma cells displayed increased GSK3β expression as opposed to BRAFi-sensitive cells. These findings were confirmed with two independent models of paired BRAFi-sensitive and resistant melanoma cells (Ma-Mel-63a, Ma-Mel-86c) [6] (Figure 1).

To confirm GSK3β upregulation and its activation also on the protein level, we employed Western blot analysis with cell lysates from BRAF-V600E melanoma cells before, during BRAFi treatment and once resistance has developed. We observed a significant increase in the activated pGSK3β in BRAFi-resistant melanoma cells when compared to BRAFi-sensitive cells. These data suggest that BRAF inhibition activates a number of pathways including the already described MEK, but, importantly, also of CREB-GSK3β (Supplementary Figures 3 and 4).

**Figure 2:**
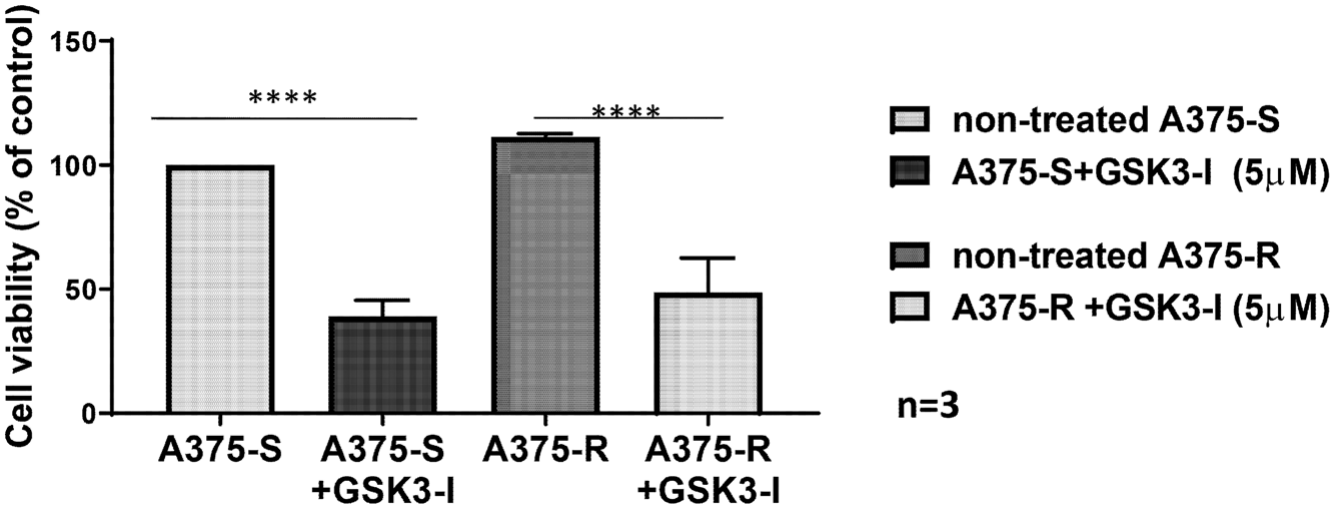
GSK inhibitor overcomes BRAF resistance. GSK3-I (LY2090314) was provided to the A375 melanoma cell line as detailed in the legend of Figure 1. GSK3-I significantly reduced the viability of BRAFi-sensitive (BRAF-S) and BRAFi- resistant (BRAF-R) melanoma cells as assessed by counting the colony-forming units employing the cell viability assay. Bars indicate % cell viability after 3 weeks of treatment with GSK-I as compared to untreated control groups. Results are given as mean ± SD. ^****^ *p* < 0.00001 by two-way ANOVA.

Remarkably, treatment of BRAFi-resistant melanoma cells with the GSK3 inhibitor LY2090314 for three weeks could overcome resistance and significantly decreased melanoma cell growth, confirming the causal role of GSK3 activation for BRAFi resistance development (Figure 2). In aggregate, our data indicate that blocking the BRAF pathway leads to an activation of GSK3β and that apart from BRAF and MEK, GSK activation constitutes an important player in cell survival during resistance development to BRAFi. Inhibitors of GSK3β reduce the cell viability of BRAFi-resistant melanoma cell lines and thus may holds promise as a novel strategy to overcome BRAFi resistance and melanoma progression.

## SUPPLEMENTARY MATERIALS


