# Supplementary material for: GSK3β activation is a key driver of resistance to Raf inhibition in BRAF mutant melanoma cells
Source: Oncotarget. 2025 Apr 4;16:257–9. doi: 10.18632/oncotarget.28711 (PMC11970936; doi:10.18632/oncotarget.28711)
Supplement: Supplementary file 1 [file oncotarget-16-28711-s001.pdf]

GSK3β activation is a key driver of resistance to Raf inhibition in BRAF mutant melanoma cells

SUPPLEMENTARY MATERIALS

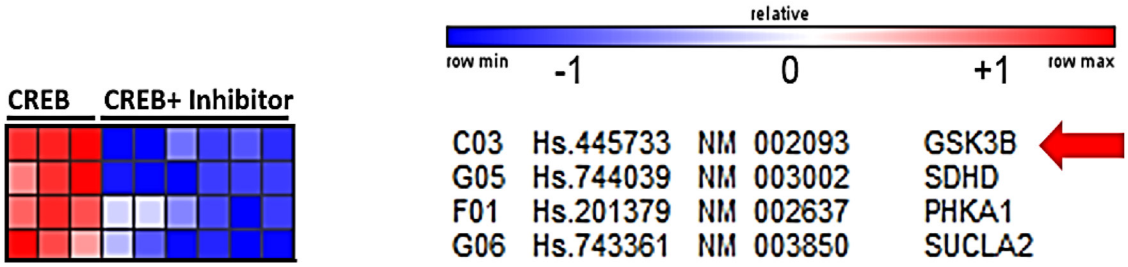

Supplementary Figure 1: CREB overexpression in metastatic BRAF-V600E mutant melanoma cells enhances the expression of GSK3β. PCR array data from BRAF-V600E A375 cells with CREB overexpression showed an increase in GSK3β expression as opposed to A375 cells with CREB overexpression treated with a CREB inhibitor (666-15, TOCRIS bioscience). This implies that the transcription factor CREB upregulates genes like GSK3β thus enforcing alternative signaling pathways involved in growth and proliferation. Upregulated genes are depicted in red color whereas downregulated genes are shown in blue. Each experimental group represents 3 replicates and was reproduced in an independent experiment. The values are represented as log2 fold change. Transcript ID and Gene ID are represented along with the gene symbol.

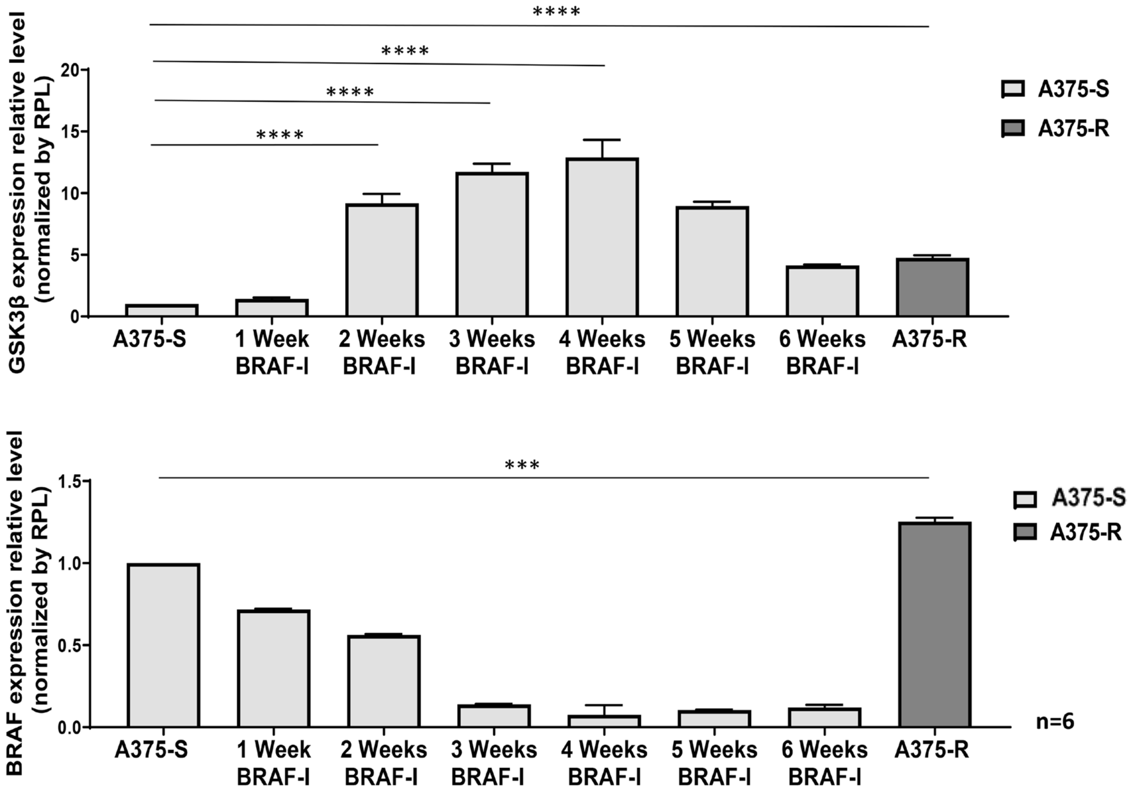

Supplementary Figure 2: GSK3β expression increases during treatment with BRAFi. qPCR data from A375 BRAF-mutated melanoma cell line treated with BRAFi depict a significant increase in GSK3β expression (upper panel) and a decrease in BRAF expression (lower panel) during BRAF inhibition for 6 weeks. At 8 weeks of BRAFi treatment, the expression of BRAF significantly increases indicative for the development of BRAF resistance. A375-S, A375 melanoma cells sensitive to BRAF-inhibition (BRAFi); A375-R, A375 melanoma cells resistant to BRAFi. DMSO served as a negative control as the BRAFi was dissolved in DMSO. Bars indicate GSK3β BRAF expression relative level normalized by RPL and expressed as mean log2 fold change ± SD. $^{*}p < 0.05$, $^{**}p < 0.001$, $^{***}p < 0.0001$, $^{****}p < 0.0001$ by one-way ANOVA.

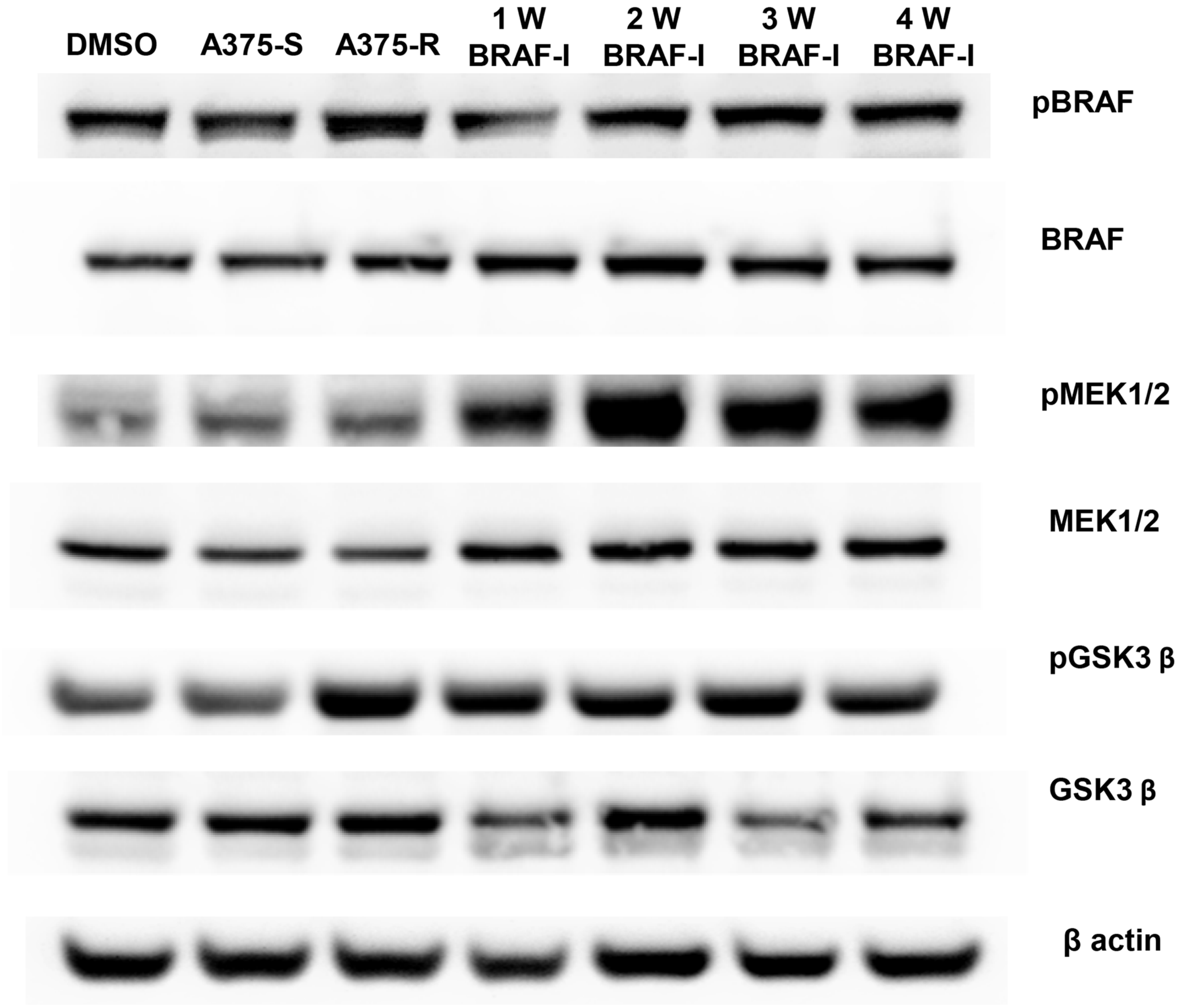

Supplementary Figure 3: Inhibition of BRAF-pathway leads to an increased expression of pGSK3β. Western blot analysis was performed on lysates from A375 melanoma cells sensitive to BRAF-inhibition (A375-S) or resistant to BRAF-inhibition (A375-R). The BRAF-inhibitor (BRAFi Dabrafenib) was employed as described in the legend of Figure 1. A substantial protein increase in activated/ phosphorylated-GSK3β was observed during therapy with the BRAFi and upon acquiring BRAF resistance. Note that also phosphorylated (activated) MEK (Mitogen-activated-protein-kinases 1/2) was observed to be upregulated in BRAFi resistant melanoma cells, a previously reported finding. An increase of activated MEK1/2 is partly responsible for BRAFi resistance. Our novel finding is that GSK3β is also increased, apparently adding to the development of BRAFi resistance. The Western blot depicts representative data reproduced in three independent experiments.

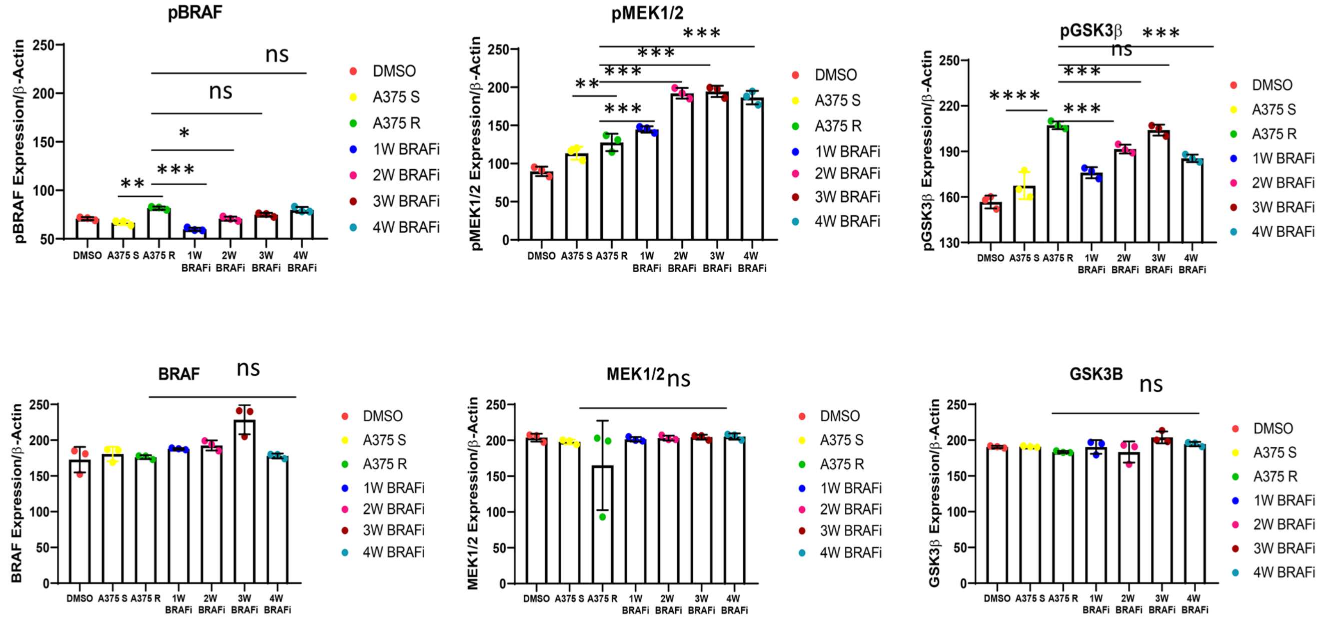

Supplementary Figure 4: pBRAF, pMEK1/2 and pGSK3β are upregulated in resistant A375 melanoma cell lines compared to A375-S. The following bar graphs represent an average densitometry data of 3 individual blots (representative blot is shown on the left panel), where groups are depicted on the x-axis and normalized protein expression ratio (Phospho or Non-phospho forms) are on the y-axis. The first group (DMSO) is the vehicle control, (A375-S) represents sensitive A375 melanoma cell line, (A375-R) represents BRAF resistant A375 melanoma cell line. (1W to 4W BRAFi) represents 1 week to 4 weeks of BRAF inhibitor treatment of A375 sensitive melanoma cells. Statistical analysis was carried out with GraphPad Prism software using One-way ANOVA and Tukey's Posthoc Test to compare means difference among groups. Significance were assessed by calculating the *P*-values as follows; $^{*}p < 0.01$, $^{**}p < 0.001$, $^{***}p < 0.0001$, $^{****}p < 0.00001$. The Non-phospho BRAF, MEK1/2, and GSK3β did not show any significant difference whereas the Phospho-form of A375-R showed a significant upregulation in pBRAF, pMEK1/2 and pGSK3β in comparison to A375-S, as well as a gradual uprising trend of phosphorylation from 1st week of BRAF inhibitor treatment reaching towards or surpassing A375-R phosphorylation levels, concluding that A375-S cells upon BRAF inhibitor therapeutic treatment in due course of time may trigger/activate alternate signaling pathways.
